# Comparison of clinical efficacy between tibial cortex transverse transport and platelet-rich plasma treatment for severe diabetic foot ulcers

**DOI:** 10.3389/fsurg.2025.1507982

**Published:** 2025-03-17

**Authors:** Pu-Xiang Zhen, Hong-Jie Su, Si-Jie Yang, Xiang Chen, Zhan-Ming Lin, Sai-Nan Liu

**Affiliations:** ^1^ National Demonstration Center for Experimental General Medicine Education, Xianning Medical College, Hubei University of Science and Technology, Xianning, China; ^2^ Department of Bone and Joint Surgery, (Guangxi Diabetic Foot Salvage Engineering Research Center), The First Affiliated Hospital of Guangxi Medical University, Nanning, Guangxi, China; ^3^ Department of Ultrasound Medicine, The Second Hospital Affiliated to Hubei University of Science and Technology, Xianning, China

**Keywords:** tibial cortex transverse transport, platelet-rich plasma, diabetic foot ulcers, stromal cell derived factor-1, wound healing

## Abstract

**Objective:**

This study aims to compare the effects of tibial cortex transverse transport (TTT) and platelet-rich plasma (PRP) on the healing of severe diabetic foot ulcers, evaluate the clinical efficacy of TTT, and explore its potential impact on lower limb circulation.

**Methods:**

A retrospective analysis was conducted on two patient groups treated at our hospital between July 2019 and June 2022. One group underwent TTT, while the other received PRP therapy. Both groups had Wagner level 3 or higher ulcers. An 18-month follow-up was performed for both groups, during which we documented wound healing progress and healing times to assess clinical efficacy. To investigate lower limb blood flow recovery, lower limb arterial ultrasound was used to measure blood flow velocities in the affected popliteal and dorsalis pedis arteries. Additionally, ELISA was employed to measure the stromal cell-derived factor-1 (SDF-1) levels of angiogenic factors in peripheral blood.

**Results:**

A total of 60 diabetic foot ulcers (DFUs) patients were enrolled in our study, with 30 patients in each group: TTT-treated and PRP-treated. During the 18-month follow-up, the wound healing rate in the TTT-treated group was significantly higher than in the PRP-treated group [96.67% (29/30) vs. 80% (24/30), *p* < 0.05]. Furthermore, the healing time in the TTT-treated group was shorter (3.02 ± 0.84 vs. 6.04 ± 0.85 months, *p* < 0.001). The amputation rate [3.33% (1/30) vs. 20% (6/30), *p* < 0.05] and recurrence rate [6.67% (2/30) vs. 26.67% (8/30), *p* < 0.05] in the TTT-treated group were lower than those in the PRP-treated group. After 1 month and 18 months of treatment, the flow velocities in the popliteal artery (68.93 ± 2.69 vs. 58.14 ± 2.48 cm/s, *p* < 0.001; 55.68 ± 3.43 vs. 46.07 ± 3.02 cm/s, *p* < 0.001) and dorsalis pedis artery (46.45 ± 2.77 vs. 36.46 ± 2.83 cm/s, *p* < 0.001; 38.63 ± 2.40 vs. 29.82 ± 2.15 cm/s, *p* < 0.001) in the TTT-treated group were significantly higher than in the PRP-treated group. Additionally, the TTT-treated group showed higher levels of SDF-1 expression (375.36 ± 13.52 vs. 251.93 ± 9.82 pg/ml, *p* < 0.001; 256.62 ± 13.19 vs. 239.96 ± 10.78 pg/ml, *p* < 0.001).

**Conclusion:**

Our results suggest that TTT treatment is more clinically effective than PRP for treating severe DFUs. This increased efficacy may be attributed to enhanced lower limb blood flow, which is potentially driven by elevated SDF-1 levels.

## Introduction

Currently, the global population of individuals with diabetes is estimated at around 425 million, with approximately one-third expected to develop diabetic foot ulcers (DFUs) (1). Shockingly, one in five DFU patients faces the risk of amputation, a condition that significantly intensifies the burden on both families and society (2).

The factors contributing to the occurrence and progression of diabetic foot ulcers (DFUs) include not only physical factors, such as long-term hyperglycemia, neuropathy, and peripheral vascular disease, but also social and cultural factors (3–5). Clinical practice employs a variety of approaches to address diabetic foot complications, including conservative drug replacement, debridement, vascular reconstruction, tendon transposition, and skin coverage (6–10). Despite these interventions, outcomes often remain unsatisfactory, particularly in severe cases of diabetic foot, where secondary infections frequently arise, exacerbating the condition and leading to amputation in a significant number of patients (10). As a result, limb preservation remains a primary objective for both patients and healthcare practitioners.

As medical technology advances, an increasing number of treatment methods are being utilized to manage diabetic foot ulcers (DFUs). One such method, tibial cortex transverse transport (TTT), has proven to be an effective treatment for severe DFUs. A clinical study involving 136 patients with severe DFUs demonstrated that after two years of follow-up, the wound healing rate reached 96%, with a recurrence rate of only 2.9% (11). TTT, a surgical approach derived from the Ilizarov technique, is based on the core principle of continuously and gradually stretching bone tissue to stimulate both local and systemic regenerative potential, thereby promoting foot wound healing. Previous studies have suggested that TTT can enhance wound healing in DFUs by improving angiogenesis and reducing local inflammation (12–14). Compared to traditional surgical approaches, this innovative technique not only promotes wound healing and limb preservation in patients with persistent diabetic foot ulcers, but also exhibits minimal postoperative complications (11, 15).

In recent years, studies have shown that platelet-rich plasma (PRP) can promote collagen synthesis, the formation of new blood vessels, and the generation of fibrous and granulation tissue by releasing biologically active substances. This, in turn, stimulates wound tissue epithelialization and accelerates tissue regeneration and repair (16). The clinical application of PRP has been expanding, now encompassing bone and soft tissue repair, chronic refractory wounds (CRWs), facial rejuvenation, and other therapeutic areas (17, 18). Numerous clinical studies, both domestically and internationally, have confirmed the positive role of PRP in the treatment of chronic wounds (19, 20).

However, there are no clinical reports comparing the efficacy of TTT and PRP in treating diabetic foot ulcers (DFUs). In this study, we retrospectively compared the wound healing outcomes of 60 DFU patients who received either TTT or PRP treatment. Additionally, we assessed the expression of stromal cell-derived factor-1 (SDF-1) factors in their peripheral blood to evaluate the clinical efficacy of these two treatment strategies.

## Methods

### Study design

The study retrospectively analyzed 60 patients with ulcerations affecting the tendon, joint capsule, or bone, who were treated at our institution between July 2019 and June 2022. One group received tibial cortex transverse transport (TTT), while the other PRP treatment. The study was approved by the hospital's Ethics Committee, and informed consent was obtained from the patients and their families (Table 1).

**Table 1:** Baseline data for patients in the TTT-treated and PRP-treated groups.

	TTT-treated (*n* = 30)	PRP-treated (*n* = 30)	*P*-value
Age (years)	64.03 ± 7.42	66.83 ± 6.51	0.73
Male sex, % (*n*)	60 (18)	63.33 (19)	0.79
BMI (kg/m^2^)	23.2 ± 3.2	23.1 ± 3.4	0.72
Duration of diabetes mellitus (years)	21.23 ± 9.61	20.56 ± 8.36	0.83
Duration of ulcers (years)	1.2 ± 0.7	1.1 ± 0.6	0.14
Ulcer area (cm^2^)	43.23 ± 9.8	40.56 ± 8.6	0.26
Wagner grading			
3	43.33 (13)	53.33 (16)	0.44
4	40.0 (12)	33.33 (10)	0.59
5	16.67 (5)	13.33 (4)	0.72
HbA1c (%)	9.6 ± 3.7	9.5 ± 3.5	0.67
Current smoker, % (*n*)	20 (6)	16.67 (5)	0.74

Data are presented as the mean ± SD or % (*n*); TTT, tibial cortex transverse transport; PRP, platelet-rich plasma.

### Setting

Inclusion criteria were as follows: patients aged >18 years, diagnosed with type II diabetes according to the American Diabetes Association criteria (21), and with foot ulcers classified as severe (grade 3 or higher) diabetic foot ulcers based on the Wagner grading system (22).

Exclusion criteria included patients with lower extremity arterial ultrasound or computed tomography angiography (CTA) results suggesting popliteal artery outflow tract stenosis >85% or occlusion; patients with psychiatric disorders unable to cooperate with surgery; patients with recent cardiovascular or cerebrovascular events, or those at high risk thereof, who were unable to tolerate surgery. We maintained detailed records of the foot ulcer's location, duration, and complications. Preoperatively, we collected wound secretions for culture to identify pathogenic bacteria and assess their antibiotic susceptibility, thereby enabling the selection of appropriate antibiotics. If the foot ulcer was open, we performed a bone probe test and a flatfoot x-ray to determine the presence of diabetic foot osteomyelitis (23). We defined peripheral arterial hypoperfusion as the absence of a palpable dorsalis pedis and posterior tibial arteries, and/or an ankle-brachial index (ABI) < 0.9 (24). Preoperatively, we assessed lower extremity vascular status using CTA. If CTA indicated severe arterial stenosis (>50% diameter reduction) or arterial occlusion due to atherosclerosis, further evaluation by a vascular surgeon and potential revascularization were required (25).

### Participants

**Figure 1:**
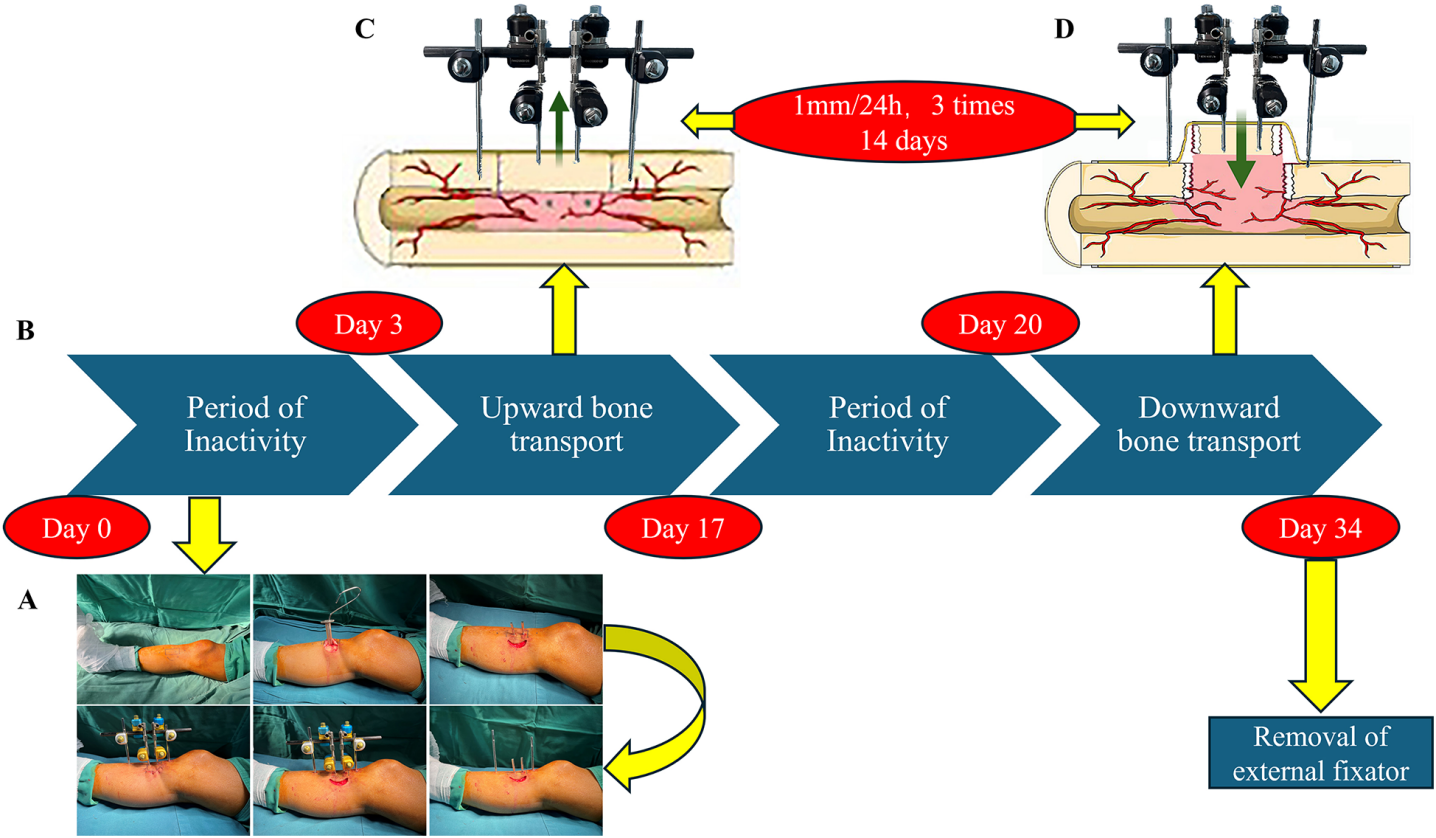
The TTT procedure and postoperative management. **(A)** TTT Surgical Steps: Initially, a fragment is excised from the medial side of the proximal tibia. This is followed by the placement of fixation pins at both ends and the center of the fragment, and the attachment of an external fixator. **(B–D)** Postoperative Process: After three days of immobilization, the patient undergoes 14 days of upward transport **(C)** and downward transport **(D)**, interspersed with three days of rest. Finally, the external fixator is removed completely.

The PRP-treated group received a PRP topical wound dressing following debridement, while the TTT-treated group underwent tibial cortex transverse transport (Figure 1). Both groups received comprehensive medical treatment to control diabetes mellitus, with prophylactic antibiotics administered intraoperatively and continued for 1 day postoperatively. Patients in the TTT-treated group underwent minimally invasive osteotomy in the operating room to create a bone fragment, and a specialized external fixator was placed under fluoroscopy.

On postoperative day 3, bone transfer was initiated using the accordion technique, performed three times daily at regular intervals, resulting in a total outward transfer of 1 mm over 2 weeks (26) Lower limb x-rays were used to confirm whether the bone fragment had reached its highest point. Following a three-day consolidation period, the 14-day reverse transport phase began. Ultimately, the bone fragment returned to its original position, which was verified by x-ray. To prevent postoperative pin tract infections, we routinely applied 75% alcohol to the pin sites. After completing the transport process, the external fixator was removed.

Patients in the PRP-treated group had 30–60 ml of peripheral venous blood collected prior to surgical debridement for PRP extraction. The blood was placed into an anticoagulation tube and centrifuged at a radius of 15 cm at 3,600 rpm for 5 min. This process removed most of the lower layer of erythrocytes and leukocytes, while retaining the plasma and platelet layer. The plasma and platelet layer were then centrifuged again for 5 min, yielding 5–10 ml of PRP. Once the necrotic tissue in the foot was debrided, the prepared autologous PRP was directly applied to the wound, and a sterile dressing was then placed over it. Subsequently, sterile dressings were changed every 7 days and covered with PRP, while monitoring the wound healing process.

### Follow up

All patients were followed up at the outpatient clinic at 4 and 12 weeks after surgery. Follow-up continued with monthly telephone calls for 18 months. The follow-up included an assessment of wound healing, recording healing time, recurrence of foot ulcers, amputation status, and any complications. In the TTT-treated group, complications included local fractures, pin tract infections, and issues arising from prolonged bed rest, such as pressure sores, pneumonia, and lower limb venous embolism. In the PRP-treated group, complications were primarily related to prolonged bed rest. To evaluate blood flow in the lower limbs, we performed lower limb arterial ultrasound to detect blood flow velocity in the affected side's popliteal and dorsalis pedis arteries. We collected peripheral venous blood preoperatively, one month postoperatively, and at the last follow-up, and assessed serum SDF-1 levels using Enzyme-Linked ImmunoSorbent Assay (ELISA). Recurrent ulcers were defined as the reoccurrence of ulcers at the same or neighboring site of a previously healed foot wound.

### Enzyme-Linked ImmunoSorbent Assay

Venous blood was collected in a serum separator tube without anticoagulants and left at room temperature for 2 h. The blood was then centrifuged at 1,000 × g for 20 min to collect the serum. Serum samples and diluted standards were added to a pre-coated ELISA plate (Abcam, ab100637), followed by incubation, washing, addition of enzyme-conjugated secondary antibody, another washing step, and color development. Absorbance at each well was measured using a microplate reader, and the SDF-1 concentration was calculated from the standard curve.

### Statistical analyses

All data were analyzed statistically using SPSS 26.0 software (Chicago, IL, USA). Continuous variables are presented as mean ± SD (for normal distribution) or median (P25, P75) (for non-normal distribution), while categorical variables are expressed as numbers and percentages. To compare the differences between two groups, the Student's t-test (for normal distribution), Mann–Whitney U test (for non-parametric variables), and chi-square test or Fisher's exact test (for categorical data) were used. Unless otherwise stated, all *p*-values were two-tailed, and a *p*-value of less than 0.05 was considered statistically significant.

## Result

### Observation of clinical efficacy

A total of 60 DFU patients were included in the study, with 30 patients in the TTT-treated group and 30 in the PRP-treated group. The baseline data of these patients are presented in Table 1. After 18 months of follow-up, the healing rate in the TTT-treated group was significantly higher than in the PRP-treated group [96.67% (29/30) vs. 80% (24/30), *p* < 0.05].

**Figure 2:**
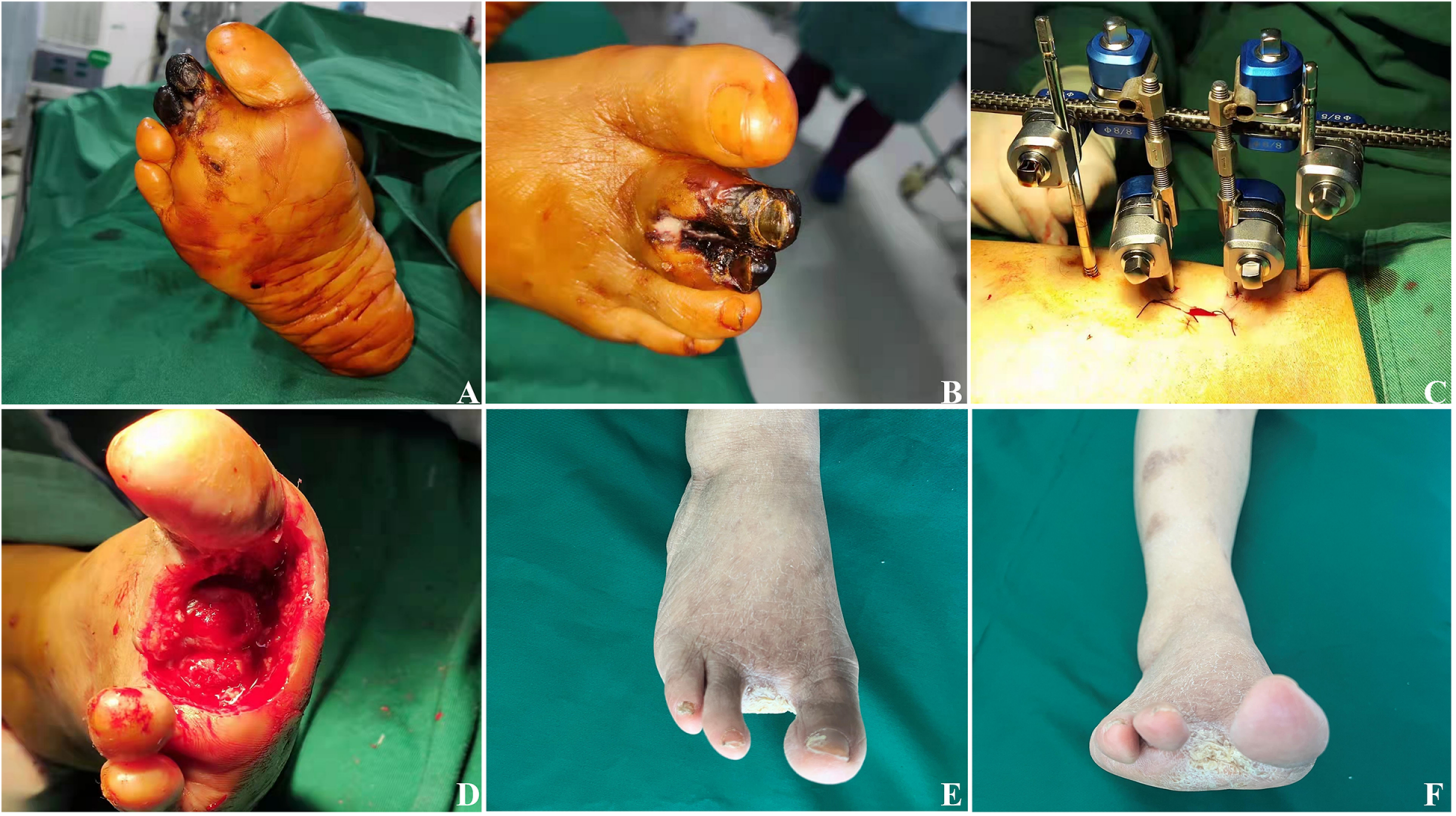
Effects of TTT surgery in a 68-year-old woman with severe and resistant plantar diabetic foot ulcer. **(A,B)** Preoperative images showing the ulcers, with evident necrosis of the second and third toes of the right foot. **(C)** An external fixator is placed on the tibia. **(D)** Intraoperative removal of the necrotic second and third toes, exposing the deep wound down to the cartilage surface. **(E)** After four weeks, the wound is nearly fully healed. **(F)** After six weeks, the original wound is completely healed.

**Figure 3:**
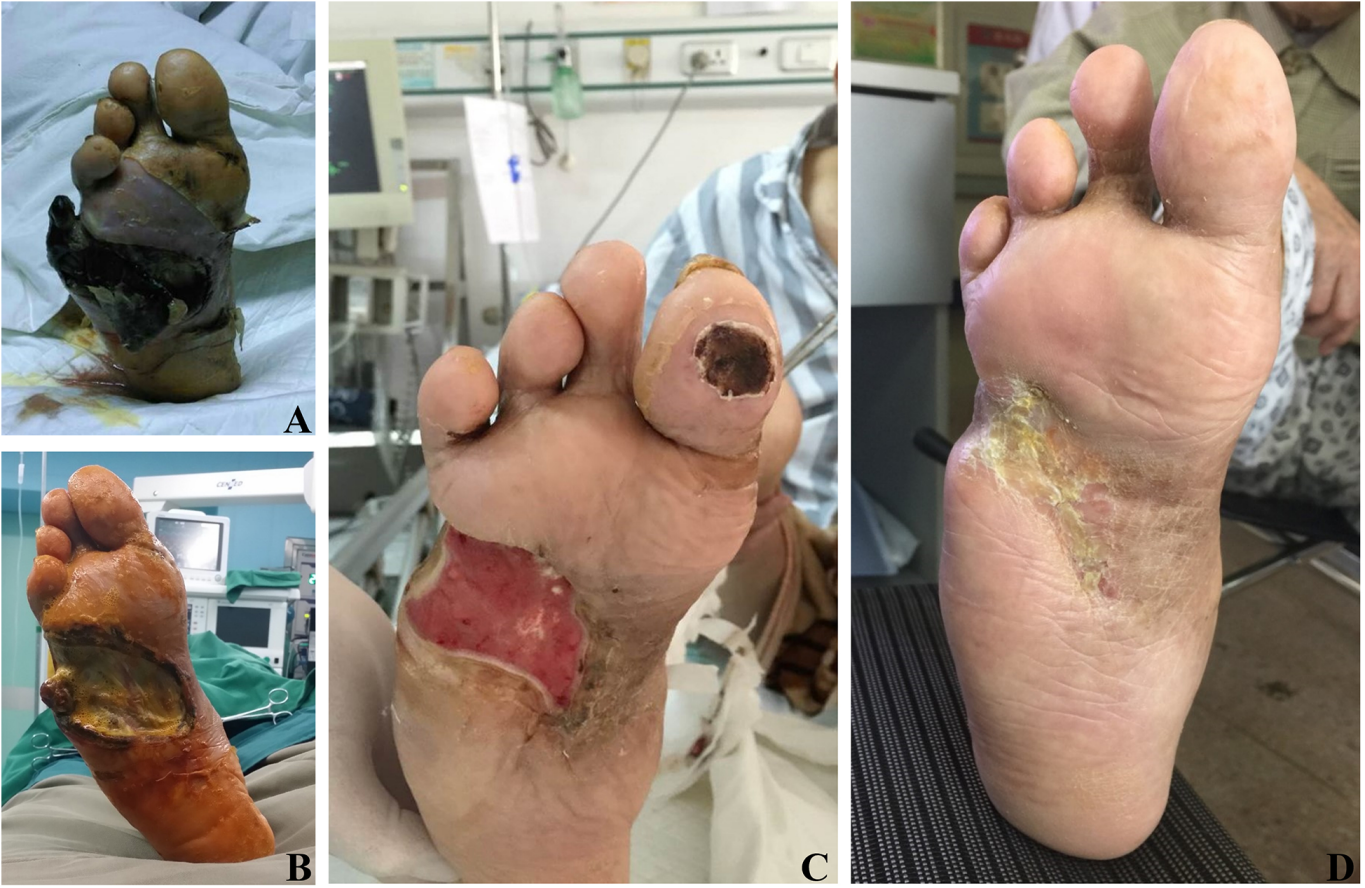
Effects of TTT surgery in a 75-year-old man with severe and resistant plantar diabetic foot ulcer. **(A)** Preoperative images showing gangrene of the fifth toe of the right foot and extensive tissue necrosis on the right plantar surface. **(B)** Ten days post-surgery, secondary tissue necrosis developed in the foot. **(C)** Four weeks post-surgery, healthy plantar granulation tissue is evident. **(D)** Eight weeks post-surgery, complete healing of the ulcers, demonstrating the effectiveness of TTT in promoting ulcer healing. Although secondary necrosis persists in the foot ulcer 10 days post-surgery, the wound continues to heal following reoperation and necrotic tissue removal.

As illustrated in Figures 2, 3, DFU patients frequently present with deep tissue infections, which underscores the importance of thorough debridement in promoting wound healing. After undergoing TTT surgery, the patient's foot wound progressed through several stages: first, soft tissue necrosis, followed by the expansion of fresh granulation tissue, and eventually reepithelialization, all culminating in complete wound healing.

**Figure 4:**
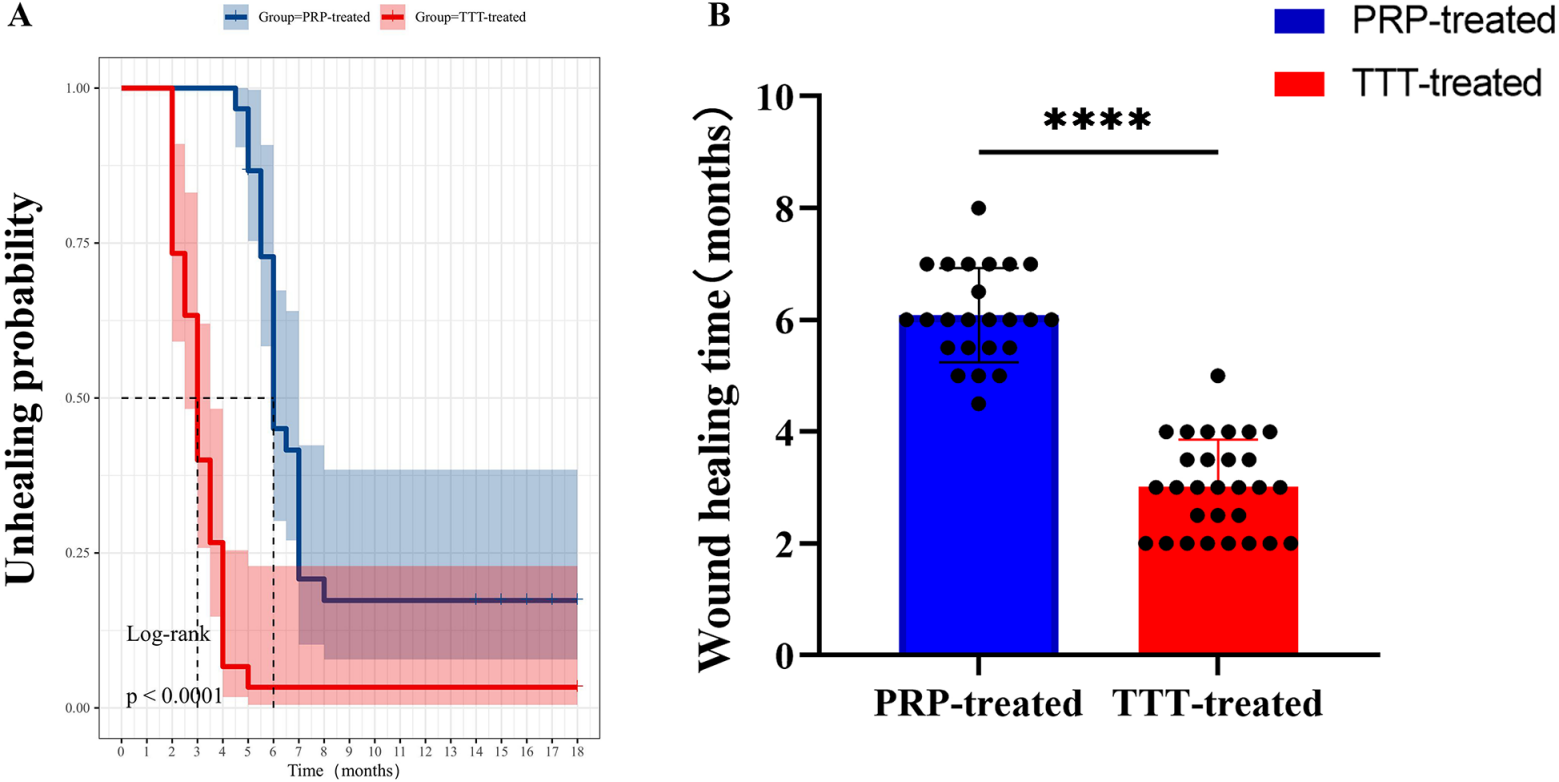
Comparison of wound healing times between the TTT-treated group and the PRP-treated group. **(A)** Kaplan–Meier curve analysis of the probability of healing in patients. **(B)** Comparison of foot healing times between the two groups of DFU patients. ****P < 0.0001.

Moreover, the average healing time in the TTT-treated group was shorter than in the PRP-treated group (3.02 ± 0.84 months vs. 6.04 ± 0.85 months, *p* < 0.001) (Figure 4). The TTT-treated group had only one patient who required amputation [3.33% (1/30)], while the PRP-treated group had six patients who underwent amputation [20% (6/30), *p* < 0.05]. Regarding foot ulcer recurrence, 2 patients in the TTT-treated group and 8 patients in the PRP-treated group experienced recurrence [6.67% (2/30) vs. 26.67% (8/30), *p* < 0.05]. Therefore, the TTT-treated group had lower amputation and ulcer recurrence rates compared to the PRP-treated group. Throughout the 18 months of follow-up, no treatment-related complications were observed in either group.

### Serum SDF-1

**Figure 5:**
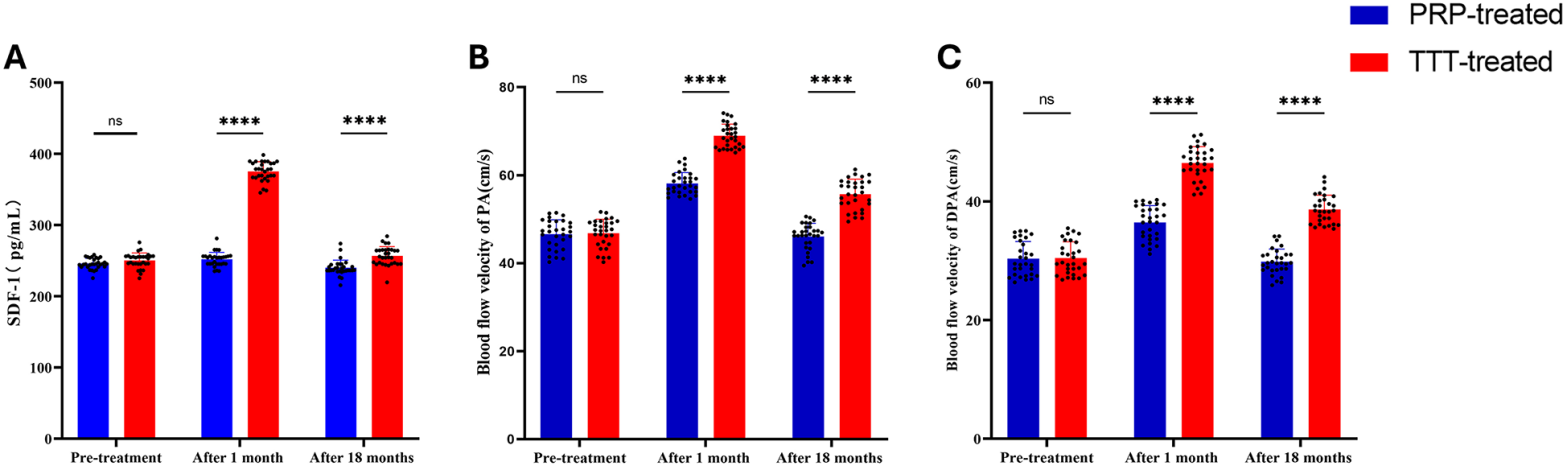
Comparison of preoperative and postoperative serum SDF-1 levels and lower limb arterial blood flow velocities between the TTT-treated and PRP-treated groups. **(A)** Serum SDF-1 concentrations. **(B and C)** Blood flow velocity in the popliteal artery **(B)** and dorsalis pedis artery **(C)** PA, popliteal artery; DPA, dorsalis pedis artery. NS, no significant difference. ****P < 0.0001.

As shown in Figure 5A, the levels of SDF-1 were not significantly different between the two groups before treatment (*P* > 0.05). After treatment, the serum levels of SDF-1 in both groups increased significantly (*P* < 0.05), with the TTT-treated group showing significantly higher levels than the PRP-treated group (375.36 ± 13.52 vs. 251.93 ± 9.82 pg/ml, *p* < 0.001). At the last follow-up, the SDF-1 level in the TTT-treated group remained higher than in the PRP-treated group (256.62 ± 13.19 vs. 239.95 ± 10.78 pg/ml, *p* < 0.001) (Table 2).

**Table 2:** Serum levels of SDF-1 in patients from the TTT-treated and PRP-treated groups (pg/ml).

	TTT-treated	PRP-treated	T-value	*P*-value
Pre-treatment	250.31 ± 10.51	245.81 ± 7.51	1.907	0.061
After 1month	375.36 ± 13.52	251.93 ± 9.82	40.456	<0.001
After 18months	256.62 ± 13.19	239.96 ± 10.78	5.358	<0.001

ELISA, Enzyme-Linked Immunosorbent Assay; TTT, tibial cortex transverse transport; PRP, platelet-rich plasma.

### Lower limb blood flow velocity

When we measured the blood flow velocity in the popliteal and dorsalis pedis arteries of both groups using a lower limb blood flow ultrasound system, no significant difference was found between the TTT-treated and PRP-treated groups before treatment (*p* > 0.05). After one month of treatment, the lower limb arterial blood flow velocity increased significantly in both groups, with the TTT-treated group showing faster velocities than the PRP-treated group (popliteal artery: 68.93 ± 2.69 vs. 58.14 ± 2.48 cm/s, *p* < 0.001; dorsalis pedis artery: 46.45 ± 2.77 vs. 36.46 ± 2.83 cm/s, *p* < 0.001). After 18 months of treatment, the lower limb blood flow velocity in both groups stabilized, but the TTT-treated group continued to exhibit faster blood flow than the PRP-treated group (popliteal artery: 55.68 ± 3.43 vs. 46.06 ± 3.02 cm/s, *p* < 0.05; dorsalis pedis artery: 38.63 ± 2.40 vs. 29.82 ± 2.15 cm/s, *p* < 0.05) (Figures 5B,C; Table 3).

**Table 3:** Blood flow velocity of popliteal artery and dorsalis pedis artery (cm/s).

		TTT-treated	PRP-treated	T-value	*P*-value
PA	Pre-treatment	46.83 ± 3.19	46.60 ± 3.28	0.282	0.779
	After 1 month	68.93 ± 2.69	58.14 ± 2.48	16.158	<0.001
	After 18 months	55.68 ± 3.43	46.07 ± 3.02	11.531	<0.001
DPA	Pre-treatment	30.45 ± 2.74	30.35 ± 2.90	0.137	0.892
	After 1 month	46.45 ± 2.77	36.46 ± 2.83	13.822	<0.001
	After 18 months	38.63 ± 2.40	29.82 ± 2,15	14.983	<0.001

PA, popliteal artery; DPA, dorsalis pedis artery.

## Discussion

Recent studies indicate that TTT is an effective treatment for DFUs, promoting ulcer healing and reducing amputation rates, which aligns with our findings (11, 27, 28). Although TTT demonstrates significant effectiveness in treating DFUs, its wider adoption has been hindered by unclear mechanisms underlying its action.

In our study, one patient in the TTT-treated group experienced a foot wound that failed to heal properly, ultimately necessitating amputation. During our follow-up, however, the residual limb healed successfully. Upon further investigation, we found that the patient had inadequate blood sugar control post-surgery and began weight-bearing on the lower limb prematurely, which led to the wound's failure to heal. Therefore, proper postoperative care and patient compliance are critical factors in ensuring the successful healing of foot ulcers.

A study indicated that TTT applies the tension-stress law, stimulating tissue regeneration (29). In animal experiments, researchers discovered that during the healing process in the fracture distraction area, the regeneration of the microvascular network occurred prior to bone tissue healing, a finding confirmed by angiography (30). Although TTT involves the manipulation of tibial fragments, this process also promotes local microvascular regeneration, which generates numerous angiogenesis-promoting substances that may positively affect the distant ulcer tissue.

Stromal cell-derived factor-1 (SDF-1), also known as CXCL12, is the exclusive ligand for the hematopoietic receptor CXCR4 and acts as a chemotactic cytokine (31). SDF-1 plays a critical role in promoting cell migration, differentiation, and vascular formation and repair (32, 33). Research indicates that activation of the SDF-1/CXCR4 axis can enhance stem cell proliferation, differentiation, and survival, while also mediating stem cell homing behavior (34). In injured tissues, the upregulation of SDF-1 recruits stem cells or progenitor cells to the damaged area, facilitating tissue repair (35). As an angiogenesis-associated factor, the increase in SDF-1 is strongly correlated with angiogenesis. In our study, we observed a significant increase in SDF-1 levels in the peripheral blood of DFU patients following TTT, along with a noticeable rise in lower limb blood flow velocity, suggesting that TTT may promote wound healing by enhancing angiogenesis in the lower limbs. A clinical study found that after TTT treatment, SDF-1 levels in the peripheral blood of DFU patients continued to rise, positively influencing angiogenesis in foot ulcers, which aligns with our findings (36).

To verify the clinical efficacy of TTT, we used PRP as a reference, as PRP is also an effective treatment for DFUs. PRP is a platelet-rich plasma concentrate obtained by centrifuging autologous whole blood, first proposed by hematologists in the 1970s as a treatment for patients with thrombocytopenia (37). PRP contains various growth factors, including platelet-derived growth factor (PDGF), transforming growth factor β (TGF-β), and insulin-like growth factor 1 (IGF-1), which are released upon activation of PRP and help promote tissue repair and regeneration (38). With further research, the applications of PRP have expanded to areas such as facial trauma, skin diseases, and facial rejuvenation (39). Due to its strong regenerative properties, PRP has been applied in the treatment of DFUs, yielding good results and significantly improving the healing rate of DFUs (40, 41). Our study results demonstrate that while PRP is effective in treating DFUs, both in terms of healing rate and healing time, TTT outperforms PRP. This is because TTT induces small artificial injuries that activate the body's regenerative potential, stimulate the generation of microvessels in the lower limbs, and promote ulcer healing.

## Limitations

This study has several limitations. First, this study was retrospective and involved a relatively small sample size, which may limit the generalizability of the results. Second, uncontrolled or unidentified confounding factors, such as the patient's age, gender, duration of illness, and presence of other comorbidities, may influence the effectiveness of the treatment. Third, the use of TTT and PRP varies across different medical institutions, potentially affecting the evaluation of treatment outcomes. Variations in standardization could account for differences in study results. Additionally, this study was not registered in the clinical trial registry for Randomized Controlled Trials (RCTs).

## Conclusion

In conclusion, we found that TTT promotes the healing of DFUs, preserves limbs, and reduces the recurrence of severe diabetic foot ulcers. Additionally, the increase in peripheral blood SDF-1 levels and enhanced lower limb blood flow suggest that the mechanism of TTT may be linked to angiogenesis. These findings indicate that TTT is an effective treatment for severe DFUs. Although no significant surgical complications were observed, further large-scale clinical trials are needed to confirm the efficacy and safety of this procedure.

## Data Availability

The original contributions presented in the study are included in the article/Supplementary Material, further inquiries can be directed to the corresponding author.
